# *Rickettsia felis*–associated Uneruptive Fever, Senegal

**DOI:** 10.3201/eid1607.100070

**Published:** 2010-07

**Authors:** Cristina Socolovschi, Oleg Mediannikov, Cheikh Sokhna, Adama Tall, Georges Diatta, Hubert Bassene, Jean-François Trape, Didier Raoult

**Affiliations:** Author affiliations: Unité de Recherche sur les Maladies Infectieuses et Tropicales Emergentes, Marseille, France (C. Socolovschi, O. Mediannikov, D. Raoult);; Unité de Recherche sur les Maladies Infectieuses et Tropicales Emergentes, Dakar, Senegal (O. Mediannikov, C. Sokhna, G. Diatta, H. Bassene, J.-F. Trape);; Institut Pasteur de Dakar, Dakar (A. Tall)

**Keywords:** bacteria, rickettsia, vector-borne infections, Rickettsia felis, Senegal, uneruptive fever, DNA extraction, children, flea-borne spotted fever, dispatch

## Abstract

During November 2008–July 2009, we investigated the origin of unknown fever in Senegalese patients with a negative malaria test result, focusing on potential rickettsial infection. Using molecular tools, we found evidence for *Rickettsia felis*–associated illness in the initial days of infection in febrile Senegalese patients without malaria.

Flea-borne spotted fever is widely distributed throughout the world (*1**,**2*). The causative agent is *Rickettsia felis,* an obligate intracellular bacterium (*2**,**3*). Usually *R. felis* infection causes mild to moderate disease characterized by fever, cutaneous rash (sometimes with an inoculation eschar), and neurologic and digestive signs (*1**–**3*). The pathogen has been detected in numerous arthropods, but the main reservoirs are *Ctenocephalides* spp. fleas, which are ectoparasites of domestic cats and dogs (*2*). Mammals that carry fleas around humans contribute to accidental infection of humans with *R. felis* after flea bites (*2**,**4*).

*R. felis* infection is generally diagnosed on the basis of both serologic assays and bacterial DNA detection by PCR (*2**–**4*). In Africa, human *R. felis* infections have been reported in Tunisia (*5*) but not in Senegal. One case of murine typhus, induced by *R. typhi,* was reported in Spain in an immigrant from Senegal (*6*). Recently, the high incidence of rickettsial diseases was noted in international travelers from sub-Saharan Africa (*7*). However, because rickettsiosis often is misdiagnosed, in the incidence of arthropod-borne spotted fever in humans in Africa may be underestimated (*4*).

The objective of our study was to investigate the origin of unknown fever in Senegalese patients who had a negative test result for malaria. We focused on potential *Rickettsia* spp. infection as a cause of fever.

## The Study

We conducted interviews and sampling during November 2008–July 2009 in 2 rural Senegalese villages in the Sine-Saloum region: Dielmo (13°43′N, 16°24′W) and Ndiop (13°41′N, 16°23′W) (*8*). The villages were included in a longitudinal prospective study, initiated in 1990, for investigations of host–parasite relationships (*8**,**9*). At the beginning of the study, all participants, including parents or legal guardians of all children, gave written individual informed consent. The national ethics committee of Senegal and the local ethics committee of Mediterranean University, Marseille, France, approved this project.

Medical examination and blood sampling were conducted for each person who had a fever >37.5°C. Approximately 200 µL (3–4 drops) of whole blood was collected from each patient by lancet stick of a fingertip for malaria tests and DNA extraction. Our study used only samples negative for *Plasmodium* spp. The first step of DNA extraction was conducted in the village dispensary by using the QIAamp kit (QIAGEN, Hilden, Germany). Binding and washing of samples with QIAGEN columns was performed with an adapted manual pump (Fisher Scientific Inc, Strasbourg, France). Columns were stored at 4°C until final elution was performed in Marseille, France.

We screened 204 samples from 134 patients by quantitative real-time PCR (qPCR) for all spotted fever group rickettsiae with *Rickettsia*-specific *gltA* gene–based RKND03 system. Positive results were confirmed by a newly designed real-time PCR primer and probe combination based on the RC0338 gene (Table 1). Appropriate handling and DNA extraction are controlled by qPCR of the β-actin gene (Table 1). We analyzed data using Epi Info software, version 3.4.1 (Centers for Disease Control and Prevention, Atlanta, GA, USA).

**Table 1:** Target sequences, primers and probes used in a study of *Rickettsia felis*, rural Senegal, November 2008–July 2009

Quantitative real-time PCR designation and specificity	Target gene	Forward primer (5′ → 3′)	Reverse primer (5′ → 3′)	Probe
RKND03 system; *Rickettsia* genus-specific	*glt*A	GTG-AAT-GAA-AGA-TTA-CAC-TAT-TTA-T	GTA-TCT-TAG-CAA-TCA-TTC-TAA-TAG-C	6-FAM-CTA-TTA-TGC-TTG-CGG-CTG-TCG-GTT-C-TAMRA
1029 system; *Rickettsia* genus-specific	Hypothetical protein (RC0338 gene)	GAM-AAA-TGA-ATT-ATA-TAC-GCC-GCA-AA	ATT-ATT-KCC-AAA-TAT-TCG-TCC-TGT-AC	6-FAM-CTC-AAG-ATA-AGT-ATG-AGT-TAA-ATG-TAA-A-TAMRA
Rpr 331 system; specific for typhus group rickettsiae	Glycosyltransferase	TGC-TTC-ATG-GGC-AAT-GTC-TG	TTG-AGC-ATA-AAA-CTG-CCC-TGC-T	6-FAM-CGC-TGG-ATT-ATC-AAA-AGA-ATT-AGC-ACG-TAMRA
Specific for *R. felis*	Biotin synthase	ATG-TTC-GGG-CTT-CCG-GTA-TG	CCG-ATT-CAG-CAG-GTT-CTT-CAA	6-FAM- GCT-GCG-GCG-GTA-TTT-TAG-GAA-TGG-G-TAMRA
β-actin; specific for human β-actin gene	Human β-actin	CAT-GCC-ATC-CTG-CGT-CTG-GA	CCG-TGG-CCA-TCT-CTT-GCT-CG	6-FAM-CGG-GAA-ATC-GTG-CGT-GAC-ATT-AAG-TAMRA

A total of 103 patients were from Dielmo (391 inhabitants), and 31 were from Ndiop (313 inhabitants). Seventy-two patients were female, and 90 (67%) were >10 years of age. No one died during the study, and all patients with identified rickettsiae infection completely recovered.

We identified 9 samples from 8 patients (6%) positive by both genus-specific qPCR systems (Table 1)*.* The following sequencing of nested PCR *gltA* gene amplicons from all positive samples showed 100% homology with *R. felis* URRWXCal2 (GenBank accession no. CP000053) (*10*). Furthermore, all positive samples were confirmed by *R. felis* species–specific qPCR. One girl 6 years age had 2 *R. felis*–positive blood samples 1.5 months apart. No samples were positive for typhus group rickettsiae, and 1 was was positive for *R. conorii* by sequencing of amplicons (data not shown).

The prevalence of flea-borne spotted fever in all tested samples was 4.4% (9/204). Monthly incidence for positive samples was 4.76% (1/21) in December, 4.76 % (2/42) in January, 16.66% (3/18) in April, 6.89% (2/29) in June, and 2.38% (1/42) in July. Seven patients lived in Dielmo, and 1 lived in in Ndiop. The overall incidence was 1.7% in Dielmo and 0.3% in Ndiop (7/391 vs. 1/313; p = 0.06), and for children <10 years of age 3.5% (5/143) in Dielmo and 0.9% (1/109) in Ndiop. The incidence of flea-borne spotted fever was highest among children <10 years of age (6/252 vs. 2/452; p = 0.02). The average age of infected patients was 15 years (range 2–57 years). Clinical manifestations are detailed in Table 2. No rashes and no eschars were found during examination.

**Table 2:** Clinical signs and symptoms in and epidemiologic data for patients with flea-borne spotted fever, rural Senegal, November 2008–July 2009*

Village	Sampling date	Age, y/sex	Temperature, °C	Signs and symptoms
Dielmo	2009 Jun	57/F	38.4	Fatigue
	2009 Apr	40/F	39.5	Chills, fatigue, headache, poor appetite, thirst, pharyngitis, sleep disorders, rhinitis, urinary pain
	2009 Jun	2/F	38	Fatigue
	2009 Jan	2/M	38.1	Cough, rhinitis,
	2009 Apr	3/M	38.1	Poor appetite, cough, sleep disorders
	2009 Jul	3/M	38.5	Headache, fatigue, poor appetite, sleep disorders
	2008 Dec	6/F	39.7	Headache
	2009 Jan		38.5	Headache
Ndiop	2009 Apr	10/F	38.7	Fatigue, headache, nausea, vomiting

*Fever was part of the case definition and therefore part of the clinical picture for all patients.

## Conclusions

Our study provides molecular evidence for *R. felis* infection in West Africa in the initial days of infection in febrile Senegalese patients who did not have malaria. This infection can be easily misdiagnosed because it lacks specific signs (*2**,**3*). We developed a 2-step DNA extraction protocol from collected whole blood. The first step, directly performed in the rural villages far from standard laboratory facilities, improved sample storage and limited contamination. Accordingly, this eliminated the need to either mount a complete DNA extraction laboratory in the field or to transport fragile samples, such as human blood, from a remote site. This method can be used for research of other infectious diseases in rural area and in other developing countries.

The major clinical signs and symptoms in our study were fever associated with weakness, headache with sleep disorders, and digestive and respiratory signs; we also noted a lack of cutaneous rash or inoculation eschar (*2**–**5*). Another rickettsial study in an indigenous African population reported that cutaneous rash might be imperceptible in patients with pigmented skin (*11*).

Interestingly, a 6-year-old child had 2 positive blood samples for *R. felis* infection; the samples were taken at 1.5 month intervals, which raises the question of potential reinfection, chronic bacteremia, or relapse. Unfortunately, the girl was not treated with antimicrobial drugs between these episodes because the samples arrived together at the laboratory for molecular diagnosis. Relapses have been described for other rickettsial diseases such as epidemic typhus with late relapse (Brill-Zinsser disease) (*12*) and scrub typhus with early relapse (*13*). Our study identified a higher attack rate of flea-borne spotted fever in children <10 years of age with an attack rate of 3.5% in Dielmo during a 9-month period. Some reports of rickettsial diseases in sub-Saharan Africa indicate more infection in younger persons in whom the disease might be mild or subclinical (*14*).

The incidence of flea-borne spotted fever was higher in Dielmo than in Ndiop. Notably, tick-borne relapsing fever, malaria, and Q fever also are more prevalent in Dielmo than in Ndiop (*8**,**9*). Reasons for the significantly different prevalence of these infectious diseases in the 2 geographically close villages remain unexplained. Our preliminary work over 9 months did not determine a seasonal variation, but we noted more cases in April. Rickettsiae, including *R. felis*, have not been reported as background organisms that may circulate undetected in the blood of humans and thus be detected by chance. Nevertheless, future work on the clarification of the role of *Rickettsia* spp. in public health will include the study of healthy controls from appropriate cohorts.

Finally, we believe that the incidence of *R. felis* infection is largely underestimated and may be responsible in Africa for many cases of uneruptive fevers of unknown origin, including those associated with respiratory, digestive, and neurologic signs. We can speculate that flea-borne spotted fever might be an important neglected public health concern not only in North Africa but also in sub-Saharan Africa. Children are particularly vulnerable to this emerging infection.
